# Identification of Potential Leukocyte Biomarkers Related to Drug Recovery of CFTR: Clinical Applications in Cystic Fibrosis

**DOI:** 10.3390/ijms22083928

**Published:** 2021-04-10

**Authors:** Marco Pedrazzi, Silvia Vercellone, Elettra Barberis, Michela Capraro, Roberta De Tullio, Federico Cresta, Rosaria Casciaro, Carlo Castellani, Mauro Patrone, Emilio Marengo, Paola Lecca, Paola Melotti, Claudio Sorio, Marcello Manfredi, Monica Averna

**Affiliations:** 1 Department of Experimental Medicine (DIMES), University of Genova, 16126 Genova, Italy; marco.pedrazzi@unige.it (M.P.); capraro36@gmail.com (M.C.); detullio@unige.it (R.D.T.); 2 Department of Medicine, General Pathology Division, University of Verona, 37134 Verona, Italy; silvyver@fastwebnet.it (S.V.); claudio.sorio@univr.it (C.S.); 3 Department of Translational Medicine, University of Piemonte Orientale, 13100 Vercelli, Italy; elettra.barberis@uniupo.it (E.B.); marcello.manfredi@uniupo.it (M.M.); 4 Cystic Fibrosis Center Genova, IRCCS Istituto Giannina Gaslini, 16147 Genova, Italy; federicocresta@gaslini.org (F.C.); RosariaCasciaro@gaslini.org (R.C.); carlocastellani@gaslini.org (C.C.); 5 Dipartimento di Scienze e Innovazione Tecnologica (DiSIT), University of Piemonte Orientale, 13100 Vercelli, Italy; mauro.patrone@uniupo.it (M.P.); emilio.marengo@uniupo.it (E.M.); 6 Faculty of Computer Science, University of Bozen-Bolzano, 39100 Bolzano, Italy; Paola.Lecca@unibz.it; 7 Cystic Fibrosis Centre, Azienda Ospedaliera Universitaria Integrata Verona, 37126 Verona, Italy; paola.melotti@aovr.veneto.it

**Keywords:** PBMCs, Ivacaftor, VX770, MMP9, proteomics, monocytes

## Abstract

The aim of this study was the identification of specific proteomic profiles, related to a restored cystic fibrosis transmembrane conductance regulator (CFTR) activity in cystic fibrosis (CF) leukocytes before and after ex vivo treatment with the potentiator VX770. We used leukocytes, isolated from CF patients carrying residual function mutations and eligible for Ivacaftor therapy, and performed CFTR activity together with proteomic analyses through micro-LC–MS. Bioinformatic analyses of the results obtained revealed the downregulation of proteins belonging to the leukocyte transendothelial migration and regulation of actin cytoskeleton pathways when CFTR activity was rescued by VX770 treatment. In particular, we focused our attention on matrix metalloproteinase 9 (MMP9), because the high expression of this protease potentially contributes to parenchyma lung destruction and dysfunction in CF. Thus, the downregulation of MMP9 could represent one of the possible positive effects of VX770 in decreasing the disease progression, and a potential biomarker for the prediction of the efficacy of therapies targeting the defect of Cl^−^ transport in CF.

## 1. Introduction

Cystic fibrosis (CF) is a multi-organ hereditary disease caused by a mutation in the gene coding for cystic fibrosis transmembrane conductance regulator (CFTR) protein. Since the discovery of the *CFTR* in 1989 [1], the search for a therapy that affects the basic defect has grown and different strategies have been undertaken. One strategy consists in the identification of small drug correctors capable of interacting with the specific mutation class molecule suitable for the type of gene mutation. To date, over 2000 different mutations in the *CFTR* have been discovered and they have been classified into six classes based on the type of defect with which the mutated protein is endowed [2,3,4,5]. The recent development of various compounds aimed to rescue CFTR function led to the registration by regulatory agencies (EMA and FDA) of Ivacaftor, a potentiator molecule (VX770) for patients carrying at least one of the following mutations: G551D, G1244E, G1349D, G178R, G551S, S1251N, S1255P, S549N or S549R [6]. Ivacaftor was the first CFTR modulator brought to the market; it is a CFTR “potentiator” that increases the amount of time the CFTR protein channel remains open. Another three CFTR modulator therapies combine Ivacaftor with one or two CFTR “correctors”: lumacaftor (VX809)/ivacaftor (LUM/IVA; Orkambi^®^) [7], tezacaftor (VX661)/ivacaftor (TEZ/IVA; Symdeko^®^ or Symkevi^®^) [8,9], and elexacaftor (VX445)/tezacaftor/ivacaftor (ELX/TEZ/IVA; Trikafta^®^ or Kaftrio^®^) [10,11]. Many other compounds are in the pipelines of biotech companies [12,13].

The clinical responses to CFTR modulators vary by genotype, and sometimes it can be difficult to predict the outcomes even within the same genotype [14,15,16]. Thus, it is important finding new biomarkers predictive of the individual patient’s response.

Cellular models that can better represent the functional features of CF include: primary bronchial epithelial cells retrieved during the lung transplantation procedure [17], nasal epithelial cells from nasal brushing [18], organoids derived from the single biopsies of intestine [19] or respiratory tissue [20], and peripheral blood leukocytes [21,22,23]. In recent years, the awareness of the significance of inflammation in CF and the finding that CFTR is expressed and has a functional impact in non-epithelial cells have raised interest in the role of immune cells in CF. CFTR defects in these cells may contribute to abnormalities in the innate immune response of CF patients [22,24], which in turn implies that the restoration of CFTR function by correctors and potentiators might be of prime importance in contributing to normalize inflammation and infection parameters in these patients. Furthermore, leukocytes can be easily and quickly isolated from patients and do not need long manipulations, and thus maintain their original features, possibly allowing to monitor the response of treatment during clinical trials [21,25] and/or test in vitro the effect of new compounds in the individual patient. In this context, a recent work concludes that the innate immune cells may act as potential surrogate markers of lung function, suggesting that the correction of the widespread dysregulation of innate immune response in CF could be an important predictive factor for the outcome of new CF therapeutics [26].

In this respect, we recently set up a method to assay CFTR activity in peripheral blood mononuclear cells (PBMCs) by using a GST-tagged iodine-sensitive yellow fluorescent protein (YFP) recombinant protein (HS-YFP assay) able to detect differential iodine transport between healthy and CF cells [23].

Shotgun proteomic analysis is a powerful technique that is able to identify and quantify thousands of proteins in a biological sample. Through the bioinformatic analysis performed on modulated proteins, the identification of pathways and biological function, as well as of new therapeutic targets, is possible. Of relevance, Braccia et al. [27] used a proteomic approach to analyze CFBE41o- cell lines and primary bronchial epithelial cells: their results showed several significantly altered protein networks, including some not previously known to be related to CF. In addition, proteomics analysis performed on CF monocytes revealed monocyte function changes in response to Ivacaftor treatment, shedding new light on the mechanism of action of the drug [28].

In this study, we monitored the activity of defective leukocyte CFTR of CF patients carrying residual function mutations eligible for Ivacaftor therapy following ex vivo treatment with VX770. Our goal was to identify, by a proteomic approach, new leukocyte biomarker(s) related to a restored CFTR activity after cell treatment with VX770 in order, not only to better understand the biological events underlying the mechanism of action of the drug, but also to predict and monitor the efficacy of therapies.

## 2. Results

We previously demonstrated that it is possible to measure CFTR activity in PBMCs isolated from human blood, and in particular, by means of the HS-YFP assay, the possibility to measure differential flows of NaI between WT and CF PBMCs [23]. However, the number of samples analyzed was quite significantly small and in this work, in order to promote this assay as a suitable method for measuring CFTR functionality in leukocytes, we first tested the activity of CFTR in a greater number of PBMCs samples and also extended this assay to monocytes directly isolated from healthy controls and CF patients.

**Figure 1:**
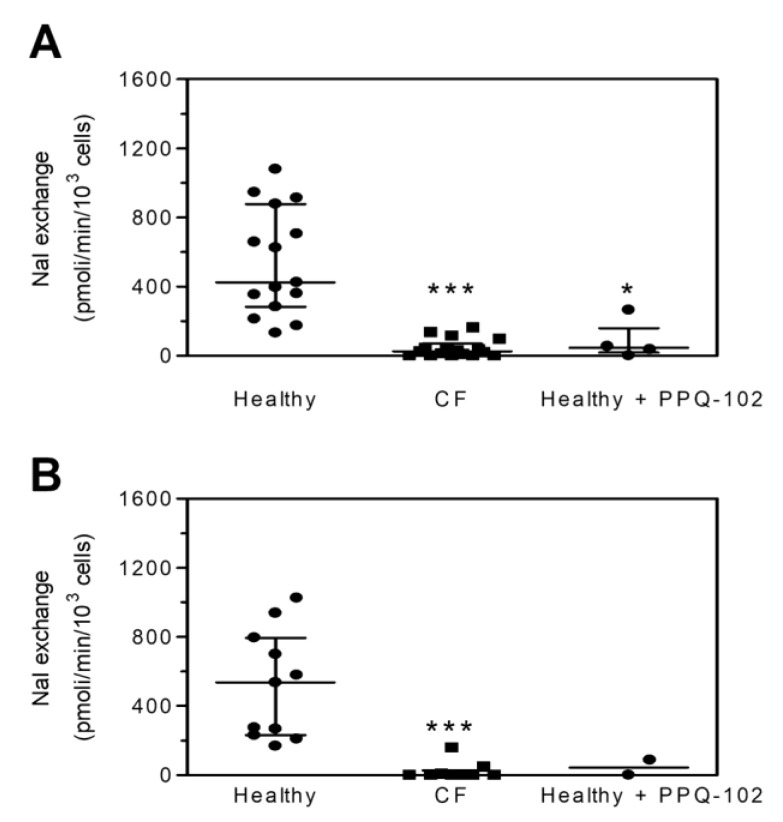
Detection of cystic fibrosis transmembrane conductance regulator (CFTR) activity in peripheral blood mononuclear cells (PBMCs). (**A**) CFTR activity was measured in PBMCs freshly isolated from healthy donors, in the absence (*n* = 15) or in the presence of 10 µM CFTR inhibitor PPQ-102 (*n* = 4), and cystic fibrosis (CF) patients F508del^+/+^ (*n* = 17). (**B**) CFTR activity was measured in monocytes isolated from healthy donors, in the absence (*n* = 11) or in the presence of 10 µM CFTR inhibitor PPQ-102 (*n* = 2), and CF patients F508del^+/+^ (*n* = 9). Data are expressed as a median with an interquartile range. Statistical analysis was carried by means of Kruskal–Wallis test followed by Dunn’s multiple comparison test. *** *p* < 0.001; * *p* < 0.05.

As indicated in Figure 1A, the median value of NaI exchange, obtained following the CFTR stimulation of healthy PBMCs, is significantly different from that obtained after the CFTR stimulation of CF PBMCs or healthy PBMCs treated with PPQ-102, a specific CFTR inhibitor. The data distribution suggests that the assay is capable of discriminating PBMCs from CF and healthy donors. Similar results were obtained with monocytes (Figure 1B), suggesting that our experimental approach could be used also with these cells. 

### 2.1. Application in Clinics

**Figure 2:**
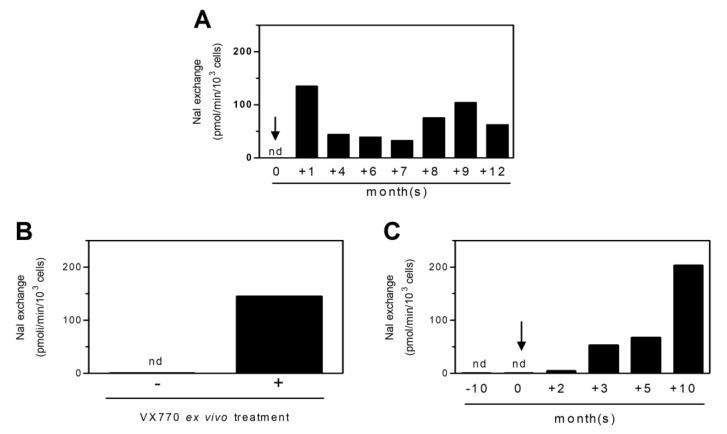
Monitoring CFTR activity following VX770 ex vivo and in vivo treatments: (**A**) CFTR activity was monitored in PBMCs freshly isolated from a CF patient carrying CFTR G1349D and F508del mutations, before (0 month) and after Ivacaftor oral therapy (+1, +4, +6, +7, +8, +9, and +12 months). nd, not detectable; (**B**) PBMCs freshly isolated from another CF patient carrying CFTR G1349D and F508del mutations were cultured in the absence (–) or in the presence (+) of 5 µM VX770. After 24 h, CFTR activity was assayed. nd, not detectable; (**C**) CFTR activity was monitored in PBMCs freshly isolated from the CF patient before (–10 and 0 months) and after Ivacaftor oral therapy (+2, +3, +5, and +10 months). nd, not detectable. The arrows indicate the beginning of the therapy.

In support of the hypothesis that the HS-YFP test on PBMCs or monocytes from CF patients could be useful in evaluating treatment efficacy, we monitored CFTR activity before and during Ivacaftor therapy in a patient carrying CFTR G1349D and F508del mutations. The NaI exchange values recorded at different times are reported in Figure 2A. Before starting the therapy, PBMCs did not show any CFTR activity (nd), but after one month of treatment, the NaI exchange values significantly increased, remaining appreciable for all intervals of monitoring.

In addition, we tested CFTR activity in the PBMCs of another patient carrying G1349D and F508del mutations before and after ex vivo treatment with VX770. As reported in Figure 2B, the cell treatment promoted a significant recovery of CFTR activity. Then, we also assayed CFTR activity in vivo before and during Ivacaftor therapy (Figure 2C). Again, no iodine exchange was detectable before therapy (nd), but after three months of treatment, the iodine exchange values significantly increased, confirming the rescue of functional CFTR due to the therapy. Altogether, these results support the possibility to assay CFTR activity on leukocytes after ex vivo treatment and the predictive value on in vivo effect.

Of interest, the readout of the HS-YFP assay from both patients related to the trend of some clinical parameters (FEV 1% and sweat chloride concentration) (Table 1).

Time indicates the months from the beginning of the Ivacaftor oral therapy. CFTR activity values correspond to those reported in Figure 2A,C, respectively.

However, the results obtained from the first patient analyzed revealed a deflection in CFTR activity, especially after 7 months of treatment. This decrease in CFTR activity paralleled with a deflection in FEV 1% from 111 to 93 (data not inserted in the table). As referred by clinicians, the deflection of FEV 1% was due to the poor drug adherence of the patient as admitted by himself. This anecdotal case suggests a good sensitivity of the assay and the possibility to also use it for monitoring adherence to therapy.

In order to monitor CFTR activity based on the effect of the only VX770, we selected a group of CF patients, carrying residual function (Supplementary Materials Table S1: *CFTR* mutations of the CF patients eligible for Ivacaftor therapy) and eligible for Ivacaftor therapy, before and after the ex vivo cell treatment with VX770. The ex vivo treatment allows to reduce the variability related to the possible outcomes of concomitant therapies occurred in vivo.

**Figure 3:**
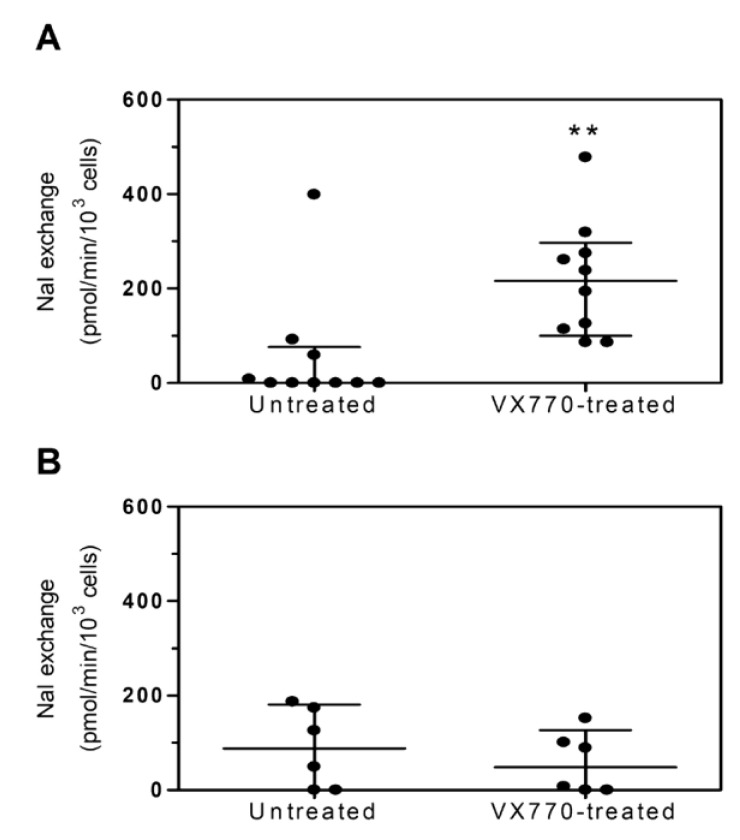
CFTR activity in PBMCs following ex vivo cell treatment with VX770. CFTR activity was measured in PBMCs isolated from CF patients (*n* = 16) and exposed for 24 h to either vehicle (untreated) or 5 µM VX770 (VX770-treated). (**A**) Responsive CF patients’ PBMCs (*n* = 10). (**B**) Unresponsive CF patients’ PBMCs (*n* = 6). Data are expressed as the median with interquartile range. Statistical analysis was carried by means of the Wilcoxon signed rank test. ** *p* < 0.01.

As shown in Figure 3, 10 of the 16 leukocyte samples analyzed for CFTR activity were responsive (Figure 3A) and six were unresponsive (Figure 3B) to the treatment with VX770 (for mutations see Supplementary Materials Table S1: *CFTR* mutations of the CF patients eligible for Ivacaftor therapy).

### 2.2. Proteomic Profile Associated to Restored CFTR Activity

**Figure 4:**
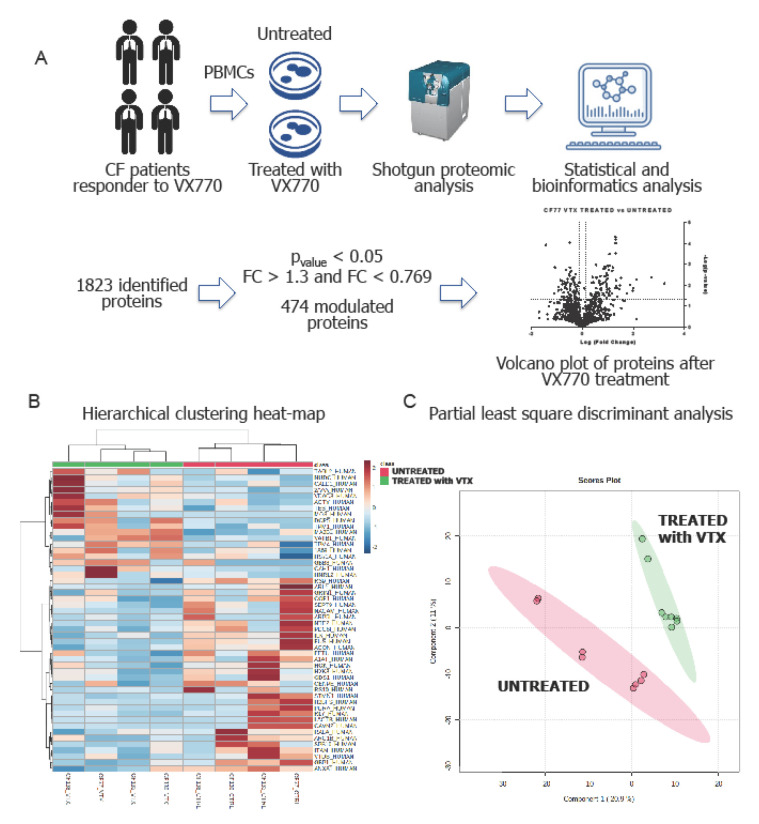
Shotgun proteomics of PBMCs treated with VX770 and untreated. (**A**) Modulated proteins were then elaborated with bioinformatics to identify biological mechanisms associated to CFTR restoration. A representative Volcano plot of four is shown. Each Volcano plot is in Supplementary Materials (Supplementary Materials Figure S1); (**B**) Clustering heat-map and (**C**) partial least square discriminant analysis (PLS-DA) of PBMCs treated with VX770 (green) and untreated (red). The heat-map was created using average proteomic values for each analyzed sample, while for the PLS-DA, technical replicates were also used.

In parallel to the evaluation of CFTR function by HS-YFP assay, we performed shotgun proteomics on PBMCs isolated from four CF patients positively responding to VX770 treatment, in order to obtain a quantitative proteomic signature directly correlated to the effect of the therapy. We used data-independent acquisition (DIA) that combines discovery proteomics with selected reaction monitoring to identify and quantify thousands of proteins. Bioinformatics analysis allowed to mine the biological pathways and functions associated to the CFTR rescue caused by the drug treatment (Figure 4A).

The proteomic analysis performed on PBMCs allowed the identification of a specific leukocyte proteomic profile composed of more than 1800 proteins (Supplementary Materials Table S2: Identified proteins). The statistical analysis based on the protein abundances reported the presence of a total of 474 modulated proteins associated to the restored CFTR activity (Supplementary Materials Table S2: Identified proteins). Multivariate analysis performed on the relative abundances of PBMCs proteins clearly showed a proteomic signature associated with the restoration of CFTR activity. The hierarchical clustering heat-map reported that VX770-treated cells (green) are well separated from untreated ones (red). The partial least square discriminant analysis confirmed the presence of structured proteomic information related to the CFTR rescue (Figure 4B,C).

To highlight the main biological functions involved in this restoration, we also performed bioinformatics analysis on differentially expressed proteins using STRING software.

**Figure 5:**
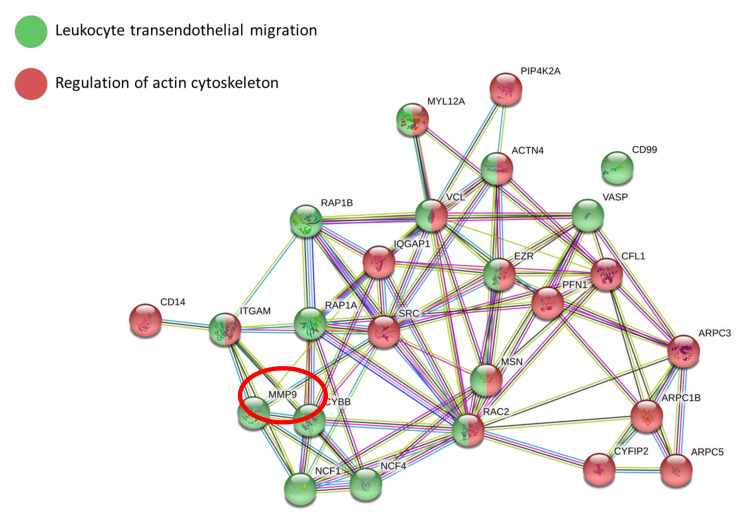
STRING network analysis of downregulated proteins in monocytes treated with VX770: the drug strongly altered leukocyte transendothelial migration and actin cytoskeleton function.

Two pathways resulted as involved after the treatment of PBMCs from all the patients. Leukocyte transendothelial migration (RAP18, RAP1A, MMP9, CYBB, NCF1, NCF4, CD99, VASP, ITGAM, RAC2, MSN, EZR, VCL, MYL12A, ACTN4) and the regulation of actin cytoskeleton (CD14, IQGAP1, SRC, PFN1, CFL1, ARPC3, ARPC1B, ARPC5, CYFIP2, PIP4K2A, RAC2, MSN, EZR, VCL, ITGAM, ACTN4, MYL12A) were downregulated after VX770 treatment (Figure 5). 

**Figure 6:**
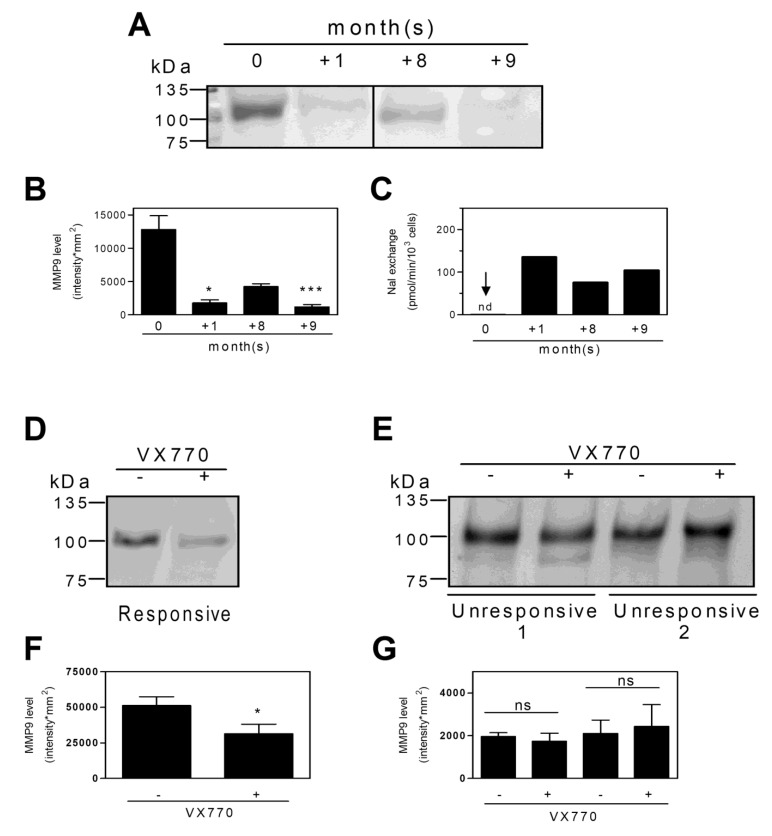
MMP9 levels in PBMCs following in vivo and ex vivo VX770 treatment: (**A**) PBMCs from CF patient carrying CFTR G1349D and F508del mutations were collected during the indicated months from the beginning of the Ivacaftor oral therapy. An aliquot of the cells was submitted to Western blot analysis for MMP9. MW protein markers (kDa) are reported. (**B**) The immunoreactive signals were quantified as specified in Methods and the data are the means ± SD from five quantifications. Statistical analysis was carried by means of a Kruskal–Wallis test followed by Dunn’s multiple comparison test. *** *p* < 0.001; * *p* < 0.05. (**C**) Selected CFTR activity values from those reported in Figure 2 for comparison to the MMP9 levels. (**D**) and (**E**) PBMCs from one responsive and two unresponsive CF patients to ex vivo VX770 treatment were submitted to Western blot for MMP9. MW protein markers (kDa) are reported. (**F**) The immunoreactive signals were quantified as specified in Methods and the data are the means ± SD from six quantifications. Statistical analysis was carried by means of Wilcoxon signed rank test. * *p* < 0.05. (**G**) The immunoreactive signals were quantified as specified in Methods and the data are the means ± SD from six quantifications. Statistical analysis was carried by means of Wilcoxon signed rank test. ns, not statistically significant.

Among the downregulated proteins, MMP9 had been previously shown by us to be upregulated in the leukocytes of F508del^+/+^ patients [29]. We confirmed the proteomic finding using Western blot in the leukocytes of a patient, whose recovery of CFTR functionality following Ivacaftor therapy is reported in Figure 2A. Figure 6 shows a strong inverse relationship between MMP9 expression and the recovery of CFTR function.

## 3. Discussion

The aim of our study was to identify specific proteomic profiles related to restored CFTR activity in CF leukocytes before and after ex vivo treatment with the potentiator VX770. To this end, we performed both the CFTR activity assay and proteomic analyses on PBMCs isolated from CF patients carrying residual function mutations and eligible for Ivacaftor therapy, before and after VX770 treatment. We took advantage of shotgun proteomics to obtain a quantitative proteomic signature directly correlated to the effect of the therapy. Moreover, thanks to the exploitation of a series of dedicated bioinformatic tools such as Ingenuity Pathway Analysis, Cytoscape, STRING, as well as multivariate analysis software such as The Unscrambler, Statistica, MatLab, we could mine the biological pathways and functions associated to CF and VX770 treatment. For this study, we decided to analyze human CF PBMCs before and after ex vivo treatment with VX770 in order to reduce the experimental variability related to the effect of possible concomitant therapy. Moreover, it is important to note that the assay of CFTR activity performed on CF PBMCs after ex vivo VX770 treatment predicted the responsiveness of the patient to Ivacaftor therapy. Thus, our experimental approach could be useful in obtaining predictive biomarkers for treatment efficacy. In addition, using the proteomic approach, we identified two leukocyte pathways, both containing proteins downregulated following treatment with VX770: leukocyte transendothelial migration and the regulation of actin cytoskeleton. A defect of monocyte adhesion, which plays a key role in immune responses and inflammation, had already been observed in patients with CF [22]. Indeed, it is reported that a defective CFTR affects chemoattractant-triggered integrin activation and chemotaxis in primary monocytes altering their trafficking in the lung parenchyma. Moreover, the involvement of actin cytoskeleton is directly correlated to the restoration of CFTR function. In fact, the interaction of CFTR with the actin cytoskeleton has been already recognized and actin disruption has been shown to both potentiate and inhibit CFTR activity depending on the experimental conditions [30,31]. In addition, Watson et al. demonstrated that CFTR’s C-terminus helps shape the actin cytoskeleton [32]. Among the downregulated proteins of the transendothelial migration pathway, we focused our interest on the protease MMP9, known to play a crucial role in CF disease progression, as reported in several prior studies [33,34,35,36,37,38]. Importantly, following secretion, MMP9 acquires not only the capability to degrade collagen, potentially contributing to parenchyma lung destruction and dysfunction, but also to potentiate chemokines involved in the modulation of inflammatory processes [39,40]. In this context, a research group recently found that the antibiotic doxycycline reduced MMP9 levels during acute CF exacerbations [33], suggesting doxycycline as an adjunctive therapy to CFTR modulators. In other studies, MMP9 has been found to be upregulated in the lower airway secretions of CF patients both in quantity and activity [38]. Furthermore, in our previous study, we found that F508del^+/+^ peripheral blood mononuclear cells also constitutively express and release MMP9 at a high rate [29], due to alterations in their intracellular Ca^2+^ homeostasis. In addition, other in vitro studies showed that CFTR modulators altered monocyte calcium homeostasis, a critical factor in initiating aberrant MMP9 secretion in CF immune cells [41]. Taken together, these evidences suggest a pathogenetic role for MMP9 overexpression present in CF leukocytes, that could contribute to worsen the airway inflammation and parenchyma lung destruction, given the recruitment of a large number of monocytes in the site of inflammation and damage [42]. In this context, as recently reported [22], an unbalanced trafficking of CF monocytes into the lung might causes the entrapment of these cells in the parenchyma and as a consequence, a persistent and prolonged secretion of MMP9 in this site. Thus, we can suggest that the downregulation of MMP9, observed in CF PBMCs treated with VX770, could represent one of the possible positive effects of this drug in decreasing lung disease progression. However, it is necessary to perform proteomic analyses also on leukocytes from non-responder patients, whose MMP9 downregulation was shown in this study by Western blot performed on only two samples. Moreover, although it has been reported that the innate immune cells may act as potential surrogate markers of lung function [26], we only investigated peripheral blood mononuclear cells and not airway immune cells. The analysis of peripheral mononuclear cells has the advantage of being useful for identifying minimally invasive biomarkers, however, in order to assess the robustness of our results, further in vivo studies are needed. Correlation of molecular data with clinical parameters such as FEV 1% and pulmonary exacerbations could be useful not only to validate predictive biomarkers of the responsiveness of the patients to new specific CFTR modulators, but also to provide knowledge about the molecular events underlying different therapy outcomes.

## 4. Materials and Methods

### 4.1. Materials

RPMI 1640, fetal bovine serum (FBS), penicillin–streptomycin solution 100X, L-glutamine 100X 200 mM, Lympholyte^®^-H, and prestained protein SHARPMASS VI MW marker were purchased from Euroclone SpA (Milan, Italy); anti-MMP9 antibody, horseradish peroxidase (HRP)-linked anti-rabbit secondary antibody, protease inhibitor cocktail (100X), and phosphatease inhibitor cocktail (100X) were obtained from Cell Signaling Technology (Danvers, MA, USA); dibutyryl-cAMP, isopropyl β-D-1-thiogalactopyranoside (IPTG), yeast extract, tryptone, PGex6P1, 6,7-Dihydro-7,9-dimethyl-6-(5-methyl-2-furanyl)-11-phenylpyrimido (4′,5′,3,4) pyrrolo (1,2-a) quinoxaline –8,10 (5H,9)-dione (PPQ-102), a reversible and voltage-independent CFTR inhibitor, and the potentiator Ivacaftor (VX770) were purchased from Sigma-Aldrich (Milan, Italy); GSH-sepharose™, ECL Select™ Western Blotting Detection Reagent, and Amersham ™ Protran^®^ Premium 0.45-μm nitrocellulose were obtained from GE Healthcare (Chicago, IL, USA); Monocytes Isolation Kit II was purchased from Miltenyi Biotec Srl (Bologna, Italy); pEYFP-C1 plasmid was obtained from Clontech Laboratories (Mountain View, CA, USA); QuickChange site-directed mutagenesis kit was from Stratagene (San Diego, CA, USA); BamHI and EcoRI restriction enzymes by Fermentas were purchased from Life Technologies Italia (Monza, Italy).

### 4.2. Ethics Statement 

All participants gave written informed consent prior to inclusion in the study, including permission to store the samples and to use them for research exclusively. The study protocol conformed to the provisions of the Declaration of Helsinki and of G. Gaslini Children Hospital, Genoa, Italy. The Ethic Committee of Genoa approved the study under protocol A-CF2014 460REG2014.

### 4.3. Donor Subjects and Sample Collection 

Our analyses were carried out on blood samples obtained from all participants during their routine clinical examinations at the hospital. Twenty-six CF patients, all F508del^+/+^ (14 females, 8 males; mean age: 38), and 26 healthy donors were enrolled for the first study (see Figure 1A,B). The CFTR assay was also performed in PBMCs isolated from two other CF patients carrying G1349D and F508del mutations (2 males; mean age 16) before and during Ivacaftor therapy. Finally, PBMCs for ex vivo treatment with VX770 were isolated from 16 CF patients carrying class III gating mutations and non-gating mutations with residual functioning CFTR, all eligible for ivacaftor therapy (8 females, 8 males; mean age: 39, see Supplementary Materials Table S1: *CFTR* mutations of the CF patients eligible for Ivacaftor therapy). All CF patients were regularly followed at the Cystic Fibrosis Center, G. Gaslini Hospital, Genova, Italy. For every patient and healthy donor, a sample of approximately 8 mL of blood was collected in three 3 mL vacuette^®^ PREMIUM tubes containing 5 mM EDTA. 

### 4.4. PBMCs and Monocytes Isolation and Cell Treatment

Blood samples were diluted with an equal volume of RPMI 1640, carefully stratified over the Lympholyte ^®^-H and centrifuged at 800× *g* for 20 min at 22 °C without brake. After centrifugation, PBMCs were collected at the interface between the upper layer, containing the plasma fraction, and the lower layer containing Lympholyte^®^-H. In order to remove platelets, PBMCs were washed twice with RPMI 1640 and then resuspended in culture medium (RPMI 1640, containing 10% (*v*/*v*) FBS, 10 U/mL penicillin, 100 µg/mL streptomycin, 2 mM L-glutamine) at 10^6^/mL cell density. To purify monocytes, we used the Monocytes Isolation Kit II (MiltenyiBiotec), following the manufacturer’s instruction. The magnetically labeled non-monocytes are depleted by retaining them on a MACS^®^ Column in the magnetic field of a MACS Separator, while the unlabeled monocytes pass through the column. After the purification, monocytes were plated in a 96-well plate without FCS and after 30 min the complete medium was added. The day after monocytes were washed twice with 20 mM sodium borate (pH 7.5), 0.25 M sucrose, 5 mM glucose, and 0.2 mM CaCl2 (CFTR Buffer) and then the assay was performed.

For ex vivo VX770-treatment, PBMCs were incubated for 24 h in the presence of the potentiator (5 µM) at 37 °C in a humidified atmosphere containing 5% CO_2_. After the incubation, PBMCs were collected, washed twice in CFTR buffer, and submitted for CFTR activity assay.

Alternatively, PBMCs were processed for either Western blot or proteomic analysis.

### 4.5. Recombinant GST-HS-YFP Purification 

The nucleotide sequence of the YFP was amplified by PCR from pEYFP-C1 plasmid with the following primers: Sn YFP-BamHI: 5′ AA-GGATCC-ATGGTGAGCAAGGGC and Asn YFP-EcoRI: 5′ A-GAATTC-TTACTTGTACAGCTCGTCCATGC. The PCR product has been cloned in pGex6P1 expression vector that contains Glutathione S-transferase (GST) as tag protein. To obtain YFP protein sensitive to halides [43] YFP-H148Q mutation was introduced. The mutation I152L that produces a YFP showing very high affinity for I-ions [44] was also introduced. Mutagenesis has been performed using the polymerase chain reaction-based QuickChange site-directed mutagenesis kit. The nucleotide sequence of the mutated YFP has been confirmed by sequencing with CEQ 2000XL DNA analysis system (Beckman Coulter).

Mutated YFP has been produced in E. Coli DH5α as GST-HS-YFP fusion protein and purified to homogeneity following affinity chromatography with GSH-Sepharose. Briefly, transformed DH5α cells were grown in Super Broth medium (3.5% tryptone, 2.0% yeast extract, 0.5% NaCl, pH 7.0) containing 100 µg/mL ampicillin at 37 °C for 16 h. Cells were then ten-fold diluted and grown at 37 °C until the optical density at 600 nm was ≥0.6. Recombinant protein expression was induced with 1.2 mM IPTG for 16 h at 25 °C. Cells were washed once with H_2_O and lysed in the following lysis buffer: 0.1 M Tris/HCl pH 8.3, 0.15 M NaCl, 1% Triton-X100, 10 mM EDTA, 2 mg/mL lysozyme, 1X Protease Inhibitor Cocktail. After 20 min at 0 °C, 10 mM MgCl_2_ and 10 µg/mL DNase were added and the lysate was incubated for an additional 20 min at 0 °C. The lysate was cleared by centrifugation (100,000× *g* for 20 min at 4 °C) and the resulting supernatant was loaded onto 2 mL GSH-sepharose column, pre-equilibrated with 50 mM sodium borate pH 7.5, containing 0.15 M NaCl and 5 mM DTT (buffer A). The resin was washed with 20 column volumes of buffer A and GST-HS-YFP was eluted with 50 mM sodium borate pH 9.0, containing 0.15 M NaCl, 5 mM DTT, and 10 mM GSH. The eluate was submitted to desalting procedure using a PD10 column (GE Healthcare) pre-equilibrated with 50 mM sodium borate pH 8.0. Purity of GST-HS-YFP was evaluated by SDS–10% PAGE followed by blue Coomassie staining.

The fusion protein has a K_I_ = 2.34 ± 0.17 (mean ± SEM) whereas HS-YFP has K_I_ = 2.05 ± 0.19 (mean ± SEM). The two K_I_ values are not statistically different according to *t* test (*p* = 0.282, for GST-HS-YFP *n* = 9 and for HS-YFP *n* = 6).

### 4.6. CFTR Assay

Measurements of CFTR activity were carried out as previously described [23]. Briefly, the cells (PBMC: 1.5 × 10^6^; monocytes: 2 × 10^5^) were stimulated with 100 µM dibutyryl-cAMP and 5 µM VX770 for 20 min at 37 °C (stimulated cells) in 200 µL of CFTR buffer. Alternatively, PBMCs (PBMCs: 1.5 × 10^6^; monocytes: 2 × 10^5^) were exposed to vehicles for 20 min at 37 °C (unstimulated cells). Hence, 5 mM NaI was added to either stimulated and unstimulated cells and after 30 s, clear supernatants were obtained by centrifugation (13,000× *g* for 20 s at room temperature). Finally, the supernatants were transferred into a black 96-well plate where 1 µg of highly purified GST-HS-YFP was added. After 5 min shaking, the fluorescence was read at λ_ex_ = 485 ± 15 nm and λ_em_ = 535 ± 10 nm. In addition, a GST-HS-YFP NaI-quenching curve was set up. Unknown NaI concentrations were extrapolated from GST-HS-YFP NaI-quenching curve, and CFTR activity was indicated as NaI exchange and expressed as pmol/min/10^3^ cells.

### 4.7. Western Blot Analysis

CF PBMCs (1.5 × 10^6^), freshly isolated as described elsewhere, were lysed by sonication in 150 μL of Laemmli sample buffer, then heated for 5 min at 95 °C, and 30 µL aliquots of each sample were separated by SDS/PAGE (8%), followed by Western blot. Nitrocellulose membrane was blocked by incubation for 1 h at room temperature with 5% skim milk powder in PBS, containing 0.05% Tween-20. Successively, the membrane was incubated for 16 h at 4 °C with primary antibodies: anti-MMP9 (1:1000). Peroxidase-conjugated secondary antibody (1 h at 22 °C) was anti-rabbit or anti-mouse (1:5000). Immunoreactive signals were developed using ECL Select ™ Western Blotting Detection Reagent, acquired and quantified using ChemiDoc ™ XRS equipped with Quantity One Image Software 4.6.1 (Bio-Rad Laboratories Srl, Segrate, MI, Italy).

### 4.8. Proteomic Analysis

The identification and quantification of the proteome modulation associated to CFTR activity was performed as previously reported [45]. Briefly, the cells were collected, washed and lysed in 1X PBS with protease inhibitors cocktail (Roche) and 0.1% SDS. Cold acetone was used for protein precipitation/denaturation followed by resuspension in 100 mM NH_4_HCO_3_. Protein content was monitored by Bradford Protein Assay (Sigma-Aldrich, St. Louis, MO). Thirty micrograms (30 µg) of proteins were subjected to reduction (with DTT from Sigma-Aldrich), alkylation (with iodoacetamide form Sigma-Aldrich), and tryptic digestion at 37 °C overnight. Peptides were then analyzed by label-free LC–MS/MS, performed by using a micro-LC system (Eksigent Technologies, Dublin, USA) interfaced with a 5600+ TripleTOF mass spectrometer (AB Sciex, Concord, Canada). Samples were subjected first to data-dependent acquisition (DDA) analysis to generate the SWATH–MS spectral library, and then to cyclic data independent analysis (DIA), based on a 25-Da window, using three technical replicates of each sample. The MS data were acquired by Analyst TF v.1.7 (AB SCIEX), while PeakView v.1.2.0.3, Protein Pilot v.4.2 (AB SCIEX) and Mascot v. 2.4 (Matrix Science) programs were used to generate the peak-list. The database search was performed using the UniProt/Swissprot (v.2015.07.07, 42131 sequences entries). Samples were input in the Protein Pilot software v. 4.2 (AB SCIEX, Concord, Canada), with the following parameters: Cysteine alkylation, digestion by trypsin, no special factors and false discovery rate at 1% were used for database search with Protein Pilot, while for Mascot search, the following parameters were used: trypsin as digestion enzyme, two missed cleavages, search tolerance of 50 ppm for the peptide mass tolerance and 0.1 Da for the MS/MS tolerance. The charges of the peptides to search for were set to 2 +, 3 + and 4 +, and the search was set on monoisotopic mass. The instrument was set to ESI-QUAD-TOF and the following modifications were specified for the search: carbamidomethyl cysteines as fixed modification and oxidized methionine as variable modification. False discovery rate was fixed at 1% [46].

The obtained files from the DDA acquisitions were used for the library generation using an FDR threshold of 1%. Protein quantification was performed by PeakView v.2.0 and MarkerView v.1.2. (ABSCIEX) programs by extracting from SWATH files ten peptides per protein with the highest MS1 intensity, and ten transitions per peptide. Peptides with FDR lower than 1.0% were exported.

### 4.9. Statistical Analysis

Where feasible, data were presented as specified in each figure legend. The significance of the difference was analyzed by non-parametric tests as indicated in the relevant figure legend, using the Prism 4.02 software package (GraphPad Software, San Diego, CA, USA), with statistical significance taken at *p* < 0.05.

Statistical analysis of proteomics data was performed using MarkerView software (Sciex) and Metaboanalyst (www.metaboanalyst.org, accessed on 9 April 2021). Proteins were considered up- and downregulated using *p*-value < 0.05 and fold change >1.3 or <0.769. Bioinformatics analysis of proteomic data was performed using STRING v.11.0 software (http://string-db.org, accessed on 9 April 2021).

### 4.10. Regression Analysis

A data analysis methodology has been implemented in the R language (www.r-project.org, accessed on 9 April 2021) to convert fluorescence intervals measured by the CFTR channel into iodide fluxes and to estimate the best mathematical model fitting these data.

The regression method is non-iterative and totally unsupervised, as it does not require an initial guess of the parameters value as input. The method implements three families of functions: polynomial (P); exponential (E); and hyperbolic functions (H). This categorization represents a large ensemble of models that can all be attributed to one of these families.

Given the (*n × m*) matrix of the experimental data, where the first column contains the measurement of the independent variable ***x*** = (*x*_1_, *x*_2_, *x_n_*), and the other columns are the arrays of the samples of the dependent variables **Y** = (Y_1_, Y_2_, Y_m_), where Y_i_ = (Y_1i_, Y_2i_, Y_ni_), the method fits the three family of functions to the data in the following way:

(1) $$\begin{array}{c} \bar{Y}={P(x)}_{\leq p}=\sum_{j=1}^{P}a_{j}x^{j}\\ \bar{Y}=E(X)=a\cdot \exp(b\cdot x)+c,\;a,b,c\in R\\ \bar{Y}=H(X)=\frac{1}{a+bx}+c,\;a,b,c\in R \end{array}$$

where $\bar{Y}=\sum_{i=1}^{m}Y_{i}$.

Finally, the model discrimination was performed on the basis of the following parameters:

- Mean sum of residuals;
- Total sum of squares;
- R-squared (where possible and reasonable to use this parameter).

The final output is the model with the lowest sum of squares, and the R-squared greater than 95%.

## Figures and Tables

**Table 1:** CFTR activity and clinical parameters during Ivacaftor oral therapy of two CF patients carrying CFTR G1349D/F508del mutation.

			Sweat Chloride (mEq/L)	
Month(s)	CFTR Activity (pmol/min/10^3^ Cells)	FEV 1 (%)	NaCl	Cl
0	0	54	93	115
+1	135	89	66	38
+8	75	105	51	28
+12	62	105	75	47
0	0	75	123	106
+3	53	122	45	22

## Data Availability

The data presented in this study are available in the article and in the Supplementary Materials.

## Supplementary Materials

The following are available online at https://www.mdpi.com/article/10.3390/ijms22083928/s1.
