# Supplementary material for: Identification of Potential Leukocyte Biomarkers Related to Drug Recovery of CFTR: Clinical Applications in Cystic Fibrosis
Source: Int J Mol Sci. 2021 Apr 10;22(8):3928. doi: 10.3390/ijms22083928 (PMC8068931; doi:10.3390/ijms22083928)
Supplement: Supplementary file 1 [file ijms-22-03928-s001.zip › FigS1.pdf]

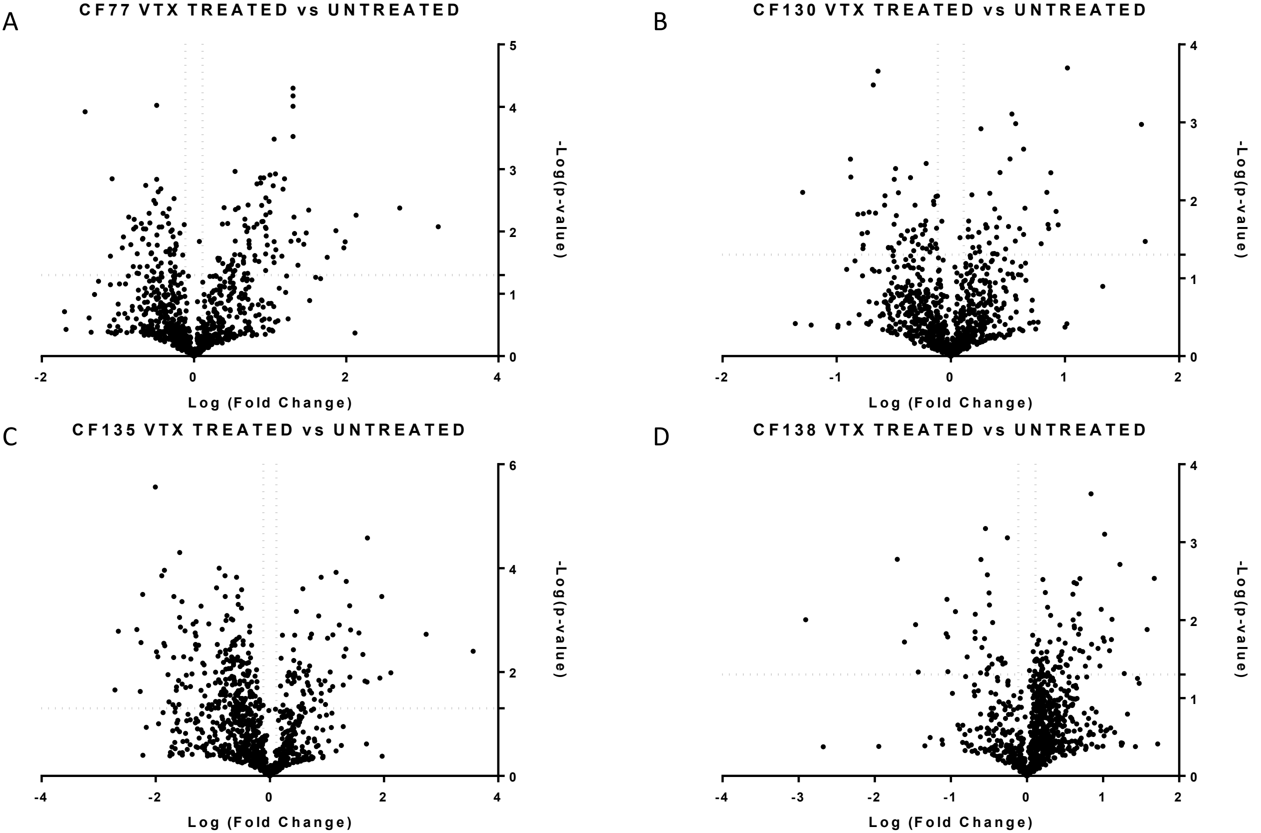
A
CF77 VTX TREATED vs UNTREATED
-Log(p-value)
Log (Fold Change)
B
CF130 VTX TREATED vs UNTREATED
-Log(p-value)
Log (Fold Change)
C
CF135 VTX TREATED vs UNTREATED
-Log(p-value)
Log (Fold Change)
D
CF138 VTX TREATED vs UNTREATED
-Log(p-value)
Log (Fold Change)
