# Supplementary material for: Identification of Potential Leukocyte Biomarkers Related to Drug Recovery of CFTR: Clinical Applications in Cystic Fibrosis
Source: Int J Mol Sci. 2021 Apr 10;22(8):3928. doi: 10.3390/ijms22083928 (PMC8068931; doi:10.3390/ijms22083928)
Supplement: Supplementary file 1 [file ijms-22-03928-s001.zip › Table S1 rev.docx]

Table S1: CFTR gene mutations of the CF patients eligible for Ivacaftor therapy.

| CFTR gene mutations | | Proteomic analysis sample ID |
| --- | --- | --- |
| 3849+10KbC>T | 1717-1G>A |  |
| 2789+5G>A | 3849+4A>G |  |
| G542X | D1152H |  |
| F508del | 2789+5G>A |  |
| G542X | D1152H |  |
| F508del | 2789+5G>A | CF135 |
| G542X | 3849+10KbC>T |  |
| F508del | 3849+10KbC>T |  |
| F508del | 3849+10KbC>T |  |
| F508del | 2789+5G>A |  |
| 1898+1G>A | D1152H |  |
| W1282X | G1244E |  |
| F508del | D1152H | CF77 |
| 457TAT>G | D1152H |  |
| F508del | 711+3A>G | CF130 |
| F508del | 711+3A>G | CF138 |

CF patients responding to VX770 *ex vivo* treatment are highlighted in pale grey. Samples submitted to proteomic analysis are indicated in the third column by the sample ID used during their processing.
